#  Attenuation of morphine tolerance and dependence by thymoquinone in mice

**Published:** 2016

**Authors:** Hossein Hosseinzadeh, Siavash Parvardeh, Alireza Masoudi, Mahsa Moghimi, Fatemeh Mahboobifard

**Affiliations:** 1 *Pharmaceutical Research Center, Department of Pharmacodynamy and Toxicology, School of Pharmacy, Mashhad University of Medical Sciences, Mashhad, Iran*; 2 *Department of Pharmacology, School of Medicine, Shahid Beheshti University of Medical Sciences, Tehran, Iran*

**Keywords:** *Thymoquinone*, *Nigella sativa*, *Morphine*, *Dependence*, *Tolerance*, *pain*

## Abstract

**Objective:**

Dependence and tolerance are major restricting factors in the clinical use of opioid analgesics. In the present study, the effects of thymoquinone, the major constituent of *Nigella sativa* seeds, on morphine dependence and tolerance were investigated in mice.

**Materials and Methods:**

Male adult NMRI mice were made tolerant and dependent by repeated injections of morphine (50, 50, and 75 mg/kg, i.p. on 9 a.m., 1 p.m., and 5 p.m., respectively) during a 3-day administration schedule. The hot-plate test was used to assess tolerance to the analgesic effects of morphine. Naloxone (2 mg/kg, i.p.) was injected to precipitate withdrawal syndrome in order to assess the morphine dependence. To evaluate the effects of thymoquinone on tolerance and dependence to morphine, different single or repeated doses of thymoquinone were administered in mice. Rotarod was used to assess the motor coordination.

**Results:**

Administration of single or repeated doses of thymoquinone (20 and 40 mg/kg, i.p.) significantly decreased the number of jumps in morphine dependent animals. Repeated administration of thymoquinone (20 and 40 mg/kg, for 3 days) and also single injection of thymoquinone (40 mg/kg, on the fourth day) attenuated tolerance to the analgesic effect of morphine. None of the thymoquinone doses (10, 20, and 40 mg/kg) produced any antinociceptive effects on their own. Motor coordination of animals was impaired by the high dose of thymoquinone (40 mg/kg).

**Conclusion:**

Based on these results, it can be concluded that thymoquinone prevents the development of tolerance and dependence to morphine.

## Introduction

Physical dependence to opioids and tolerance to their analgesic effects, are major problems limiting the clinical use of opioids (Alldredge et al., 2013 ▶). To overcome tolerance and dependence to opioid analgesics, many attempts have been made using strategies focused on the underlying mechanisms. In this regard, several drugs and natural compounds have been investigated for their possible inhibitory effects on opioid dependence and tolerance. Among them, medicinal herbs have attracted considerable interest by scientists in the last two decades. 

Several studies have been shown that applying herbal extracts/oils may be beneficial in the attenuation of dependence and tolerance to opioid agents (Tabatabai et al., 2014 ▶; Han et al., 2014 ▶; Mansouri et al., 2014 ▶). Among numerous medicinal herbs, *Nigella sativa* L. (*N. Sativa*), a member of the Ranunculaceae family, is emerging as a unique herb with outstanding therapeutic effects. *N. sativa*, has been traditionally used for the treatment of respiratory, gastrointestinal, kidney, and liver diseases. Many medicinal properties of *N. sativa* have been attributed to its seeds extracts and/or oil (Ziaee et al., 2012 ▶; Ahmad et al., 2013 ▶; Tembhurne et al., 2014 ▶). Recently, it has been shown that *N. sativa* possess analgesic (Bashir and Qureshi, 2010 ▶), anti-inflammatory (Bourgou et al., 2012 ▶), antioxidant (Bourgou et al., 2012 ▶; Leong et al., 2013 ▶; Sultan et al., 2014 ▶; Cikman et al., 2014 ▶; Develi et al., 2014 ▶), antidiabetic (Sultan et al., 2014 ▶ Mathur et al., 2011 ▶), antihypertensive (Leong et al., 2013 ▶), antibacterial (Hosseinzadeh et al., 2007 ▶; Bourgou et al., 2012 ▶), neuroprotective (Ezz et al., 2011 ▶; Hobbenaghi et al., 2014 ▶), antiepileptic (Ezz et al., 2011 ▶; Bhandari, 2014 ▶), antiasthmatic and bronchodilatory (Boskabady et al., 2004 ▶, 2010, 2011), protective effects on the kidney and adrenal gland (Babazadeh et al., 2012 ▶; Dollah et al., 2013 ▶), and antineoplastic effects (Al-Sheddi et al., 2014 ▶; Norfazlina et al., 2014 ▶). 

Many medicinal properties of *N. sativa* seeds extracts and/or its oils, are attributed to a quinonic compound called thymoquinone. It has been shown that thymoquinone has several pharmacological properties such as antioxidant (Khan et al., 2012 ▶; Jrah-Harzallah et al., 2012 ▶, 2013), anticonvulsant (Hosseinzadeh and Parvardeh, 2004 ▶; Hosseinzadeh et al., 2005 ▶; Akhondian et al., 2011 ▶), antinociceptive (Abdel-Fattah et al., 2000 ▶), and neuroprotective effects (Hosseinzadeh et al., 2007 ▶; Ismail et al., 2013 ▶; Radad et al., 2014 ▶). 

Recently, it has been reported that *N. sativa* oil attenuated tolerance and dependence induced by morphine and tramadol in mice (Abdel-Zaher et al., 2010 ▶, 2011). Since thymoquinone is the major constituent of *N. sativa* oil, it can be hypothesized that the inhibitory effects of *N. sativa* on opioid-induced tolerance and dependence may arise from the neuroprotective effects of thymoquinone. Thus, the present study was carried out in order to clarify the effects of thymoquinone, the major constituent of *N. sativa* seeds, on the development of physical dependence, the expression of withdrawal syndrome, and the development of tolerance to analgesic effects of morphine in mice.

## Materials and Methods

**Animals**

Adult male NMRI mice weighing 20-30 g were obtained from the animal house of Shahid Beheshti University of Medical Sciences (Tehran, Iran). The animals were housed in plastic cages and kept at 23±2 °C on a 12 hours light/dark cycle at least 7 days prior to testing. Commercial food pellets and tap water were freely available at all times except during the experiments.

All procedures and experiments were carried out in accordance with institutional guidelines for laboratory animal care and use.

**Drugs**

The following drugs were used in this study: thymoquinone (Sigma-Aldrich), morphine sulphate (Temad, Iran), naloxone hydrochloride (Tolidaru, Iran), and diazepam (Caspian, Iran). Drugs were dissolved in normal saline. Thymoquinone was suspended in 0.8% (v/v) tween 80. All compounds were prepared freshly and administered intraperitoneally (i.p.) in a volume of 0.1 ml/20 g body weight. Control animals received the same volume of vehicle (normal saline + tween 80). 

**Induction of dependence and tolerance**

In order to induce dependence and tolerance in mice, morphine was administered intraperitoneally (i.p.) 3 times per day at 9 a.m. (50 mg/kg), 1 p.m. (50 mg/kg), and 5 p.m. (75 mg/kg) for 3 consecutive days. The dose of 75 mg/kg at 5 p.m. was administered to prevent overnight withdrawal syndrome in animals. The last dose of morphine (50 mg/kg) was administered on the fourth day at 9 a.m. (Zarrindast and Torkaman-Boutorabi, 2003 ▶). Hyperactivity and Straub-tail reaction were seen after morphine injections in mice. Weight loss and death were recorded during the chronic administration of morphine.

**Evaluation of dependence to morphine**

The animals were divided into nine groups (n=7 each): Group 1 (negative control) received morphine and vehicle (normal saline + tween 80). Groups 2, 3, and 4 received morphine and different single doses of thymoquinone (10, 20, and 40 mg/kg, respectively) on day 4. Groups 5, 6, and 7 received morphine and different doses of thymoquinone (10, 20, and 40 mg/kg, respectively) three times per day on days 1, 2, and 3, but not on day 4. Groups 8 and 9 (positive controls) received morphine and diazepam (4 mg/kg).

Two hours after the injection of the last dose of morphine on the fourth day, naloxone (2 mg/kg, i.p.), the selective antagonist of opioid receptors, was injected to the animals (groups 1-9) to precipitate withdrawal syndrome. Mice were immediately placed individually in a plexiglass box and the number of jumps, as the major sign of withdrawal syndrome, was recorded during a 30-min period (Zarrindast and Torkaman-Boutorabi, 2003 ▶). 

To evaluate the effect of thymoquinone on the expression of physical dependence which is determined by the signs of withdrawal syndrome, three different single doses of thymoquinone (10, 20, and 40 mg/kg, i.p.) were administered 30 min before the injection of naloxone (2 mg/kg, i.p.) in mice on the fourth day. These animals (groups 2-4) did not receive thymoquinone before the injection of morphine during the 3-day administration schedule.

In order to assess the effects of thymoquinone on the induction of physical dependence to morphine, different doses of thymoquinone (10, 20, and 40 mg/kg, i.p.) were administered in mice 30 min before the injection of morphine during the 3-day administration schedule. These animals (groups 5-7), did not receive thymoquinone on the fourth day.

**Evaluation of tolerance to morphine**

The animals were divided into seven groups (n=7 each): Group 10 (control) received morphine and vehicle (normal saline + tween 80). Groups 11, 12, and 13 received morphine and different single doses of thymoquinone (10, 20, and 40 mg/kg, respectively) on day 4. Groups 14, 15, and 16 received morphine and different doses of thymoquinone (10, 20, and 40 mg/kg, respectively) three times per day on days 1, 2, and 3, but not on day 4. Moreover, the analgesic effect of different single doses of thymoquinone (10, 20, and 40 mg/kg) was evaluated on 3 more groups of mice (groups 17-19) to determine whether or not, thymoquinone possesses antinociceptive effect on their own. These animals did not receive any other drugs.

In order to assess the development of tolerance to analgesic effects of morphine in control or thymoquinone-treated mice, the hot-plate test was used. Thirty minutes after the injection of morphine (10 mg/kg, i.p.) on the days 1 and 4, the mice were placed on a metal plate maintained at 55±1 °C and the latency of nociceptive responses including licking or flicking of the limbs or jumping were measured in the animals. A cut-off time of 45 s was selected to prevent tissue damage. The latency of nociceptive responses in mice was expressed as the hot-plate latency (Vogel, 2008 ▶).

To evaluate the effect of thymoquinone on the expression of tolerance to morphine, three different single doses of thymoquinone (10, 20, and 40 mg/kg, i.p.) were administered 30 min before the injection of the last dose of morphine (10 mg/kg, i.p.) on the fourth day. These animals (groups 11-13) did not receive thymoquinone before the injection of morphine during the 3-day administration schedule. Moreover, to assess the effects of thymoquinone on the development of morphine tolerance, different repeated doses of thymoquinone (10, 20, and 40 mg/kg, i.p.) were administered 30 min before the injection of morphine during the 3-day administration schedule. These animals (groups 14-16) did not receive thymoquinone before the injection of morphine on the fourth day. 

**Evaluation of motor coordination**

The effects of thymoquinone and diazepam on motor coordination was assessed using the rotarod apparatus (RotaRod 3375-5, TSE System). In this test, separate groups of animal were used. On the first day, mice were trained to remain for 5 min on a rod rotating at the initial and final speed of 10 and 20 rpm, respectively (the acceleration time was 20 sec). On the next day, 60 and 90 min after the administration of the different doses of thymoquinone (10, 20, and 40 mg/kg, i.p.) or diazepam (4 mg/kg, i.p.), the animals were placed on the rotating rod and the length of time each animal remained on the rotating rod (time-on-rod) was recorded (Hosseinzadeh and Parvardeh, 2004). In the evaluation of motor coordination, none of the animals received morphine. 

**Statistical analysis**

The data were expressed as mean values ± S.E.M for 7 mice and tested with analysis of variance (ANOVA) followed by the multiple comparison test of Tukey. Differences between means were considered statistically significant if p<0.05. 

## Results

**Effects of thymoquinone on morphine dependence**

Repeated administration of morphine induced physical dependence in mice in control group. These animals (group 1) received vehicle (normal saline + tween 80) 30 min before each dose of morphine during the 3-day administration schedule. Administration of naloxone (2 mg/kg, i.p.) two hours after the injection of the last dose of morphine (50 mg/kg, i.p.) on the fourth day, precipitated the withdrawal syndrome in control group so that the jumping behavior was significantly increased in mice (Tables 1, 2). 

Administration of single doses of thymoquinone (20 and 40 mg/kg, i.p.) 30 minutes before the injection of naloxone (2 mg/kg, i.p.) on the fourth day, significantly decreased the number of jumps in morphine-dependent animals in groups 3 and 4 (p<0.05 and p<0.001, respectively) (Table 1). Moreover, repeated administration of thymoquinone at the doses of 20 and 40 mg/kg (groups 6 and 7), 30 minutes before the injection of morphine during the 3-day administration schedule (but not at the fourth day), significantly decreased the number of jumps in morphine-dependent mice (p<0.05 and p<0.01, respectively) (Table 2). Diazepam (4 mg/kg, i.p.), as the positive control, significantly attenuated withdrawal syndrome in morphine-dependent mice (Tables 1, 2). Lower dose of thymoquinone (10 mg/kg) did not have any effect on the expression (group 2) or induction (group 5) of physical dependence to morphine in mice (Tables 1, 2). Loss of weight (5-15%) and death (1-2%) occurred with chronic administration of morphine sulfate.

**Table 1:** Effects of thymoquinone on the expression of morphine dependence in mice

**Drug (dose)**	**Number of jumps**
**Control**	45.2 ± 10.7
**Diazepam (4 mg/kg)**	3.0 ± 0.4 ***
**Thymoquinone (10 mg/kg)**	31.8 ± 9.5
**Thymoquinone (20 mg/kg)**	17.4 ± 3.8 *
**Thymoquinone (40 mg/kg)**	10.0 ± 2.7 **

Thymoquinone and controls were administered 30 min before the injection of each doses of morphine during the 3-day administration schedule (but not on the fourth day). Naloxone (2 mg/kg, i.p.) was administered 2 hours before the injection of the last dose of morphine (50 mg/kg, i.p.) on the fourth day. Control: Normal saline + Tween 80 (0.8%, v/v); Values are the mean ± SEM for 7 mice;

* p<0.05,

** p<0.01,

*** p<0.001, as compared to control.

**Table 2:** Effects of thymoquinone on the induction of morphine dependence in mice

**Drug (dose)**	**Number of jumps**
**Control**	42.6 ± 9.4
**Diazepam (4 mg/kg)**	2.1 ± 0.2 ***
**Thymoquinone (10 mg/kg)**	27.0 ± 3.3
**Thymoquinone (20 mg/kg)**	16.4 ± 5.2 *
**Thymoquinone (40 mg/kg)**	0.3 ± 0.3 ***

Thymoquinone and controls were administered 30 min before the injection of naloxone (2 mg/kg, i.p.) on the fourth day. Control: Normal saline + Tween 80 (0.8%, v/v); Values are the mean ± SEM for 7 mice;

* p<0.05,

*** p<0.001, as compared to control.

**Effects of thymoquinone on morphine tolerance**

Analgesic effects of morphine following repeated administration during 3 days, decreased significantly in the hot-plate test (p<0.001). However, repeated administration of thymoquinone (20 and 40 mg/kg, i.p.) 30 minutes before the injection of morphine during 3 days, significantly attenuated the development of tolerance to analgesic effects of morphine in groups 15 and 16 (p<0.05 and p<0.01, respectively) (Table 3).

**Table 3:** Effects of thymoquinone on the induction of tolerance to analgesic effects of morphine in mice

**Drug (dose)**	**Hot-plate latency (s)**
**Control**	14.48 ± 1.62
**Morphine (10 mg/kg), day 1**	34.35 ± 3.47 ***
**Morphine (10 mg/kg), day 4**	9.68 ± 1.29
**Thymoquinone (10 mg/kg)**	15.14 ± 1.95
**Thymoquinone (20 mg/kg)**	19.75 ± 3.05*
**Thymoquinone (40 mg/kg)**	22.24 ± 2.96 **

Thymoquinone and control were administered intraperitoneally 30 min before the injection of morphine during the 3-day administration schedule. The last dose of morphine (10 mg/kg, i.p.) was administered on the fourth day. The animals did not received thymoquinone or control on the fourth day. Control: Normal saline + Tween 80 (0.8%, v/v); Values are the mean ± SEM for 7 mice;

* p<0.05,

** p<0.01,

*** p<0.001, as compared to control;

p<0.05,

p<0.01, as compared to morphine day 4.

Moreover, administration of single dose of thymoquinone (40 mg/kg, i.p.) 30 minutes before the injection of the last dose of morphine (10 mg/kg, i.p.) on the fourth day, prolonged the latency of nociceptive response in hot-plate test (group 13, p<0.01) (Table 4). However, thymoquinone at the doses of 10 and 20 mg/kg (groups 11 and 12, respectively) did not have any effect on the tolerance to analgesic effects of morphine (Table 4).

The attenuating effect of thymoquinone on morphine tolerance was not attributed to the analgesic effects of thymoquinone, because none of the thymoquinone doses (10, 20, and 40 mg/kg) produced any antinociceptive effects on their own (Table 5). 

**Table 4.** Effects of thymoquinone on the expression of tolerance to analgesic effects of morphine in mice

**Drug (dose)**	**Hot-plate latency (s)**
**Control**	14.48 ± 1.62
**Morphine (10 mg/kg), day 1**	34.35 ± 3.47 ***
**Morphine (10 mg/kg), day 4**	9.68 ± 1.29
**Thymoquinone (10 mg/kg)**	10.22 ± 1.98
**Thymoquinone (20 mg/kg)**	17.32 ± 2.35
**Thymoquinone (40 mg/kg)**	24.27 ± 4.17 **

Thymoquinone and control were administered intraperitoneally 30 minutes before the injection of the last dose of morphine (10 mg/kg, i.p.) on the fourth day. Control: Normal saline + Tween 80 (0.8%, v/v); Values are the mean ± SEM for 7 mice;

** p<0.01,

*** p<0.001, as compared to control;

p<0.01, as compared to morphine day 4.

**Table 5:** Effects of thymoquinone on nociceptive responses of mice in hot-plate test

**Drug (dose)**	**Hot-plate latency (s)**
**Control**	14.48 ± 1.62
**Thymoquinone (10 mg/kg)**	13.78 ± 0.89
**Thymoquinone (20 mg/kg)**	13.01 ± 0.84
**Thymoquinone (30 mg/kg)**	12.71 ± 0.98

Thymoquinone and control were administered intraperitoneally 30 min before placing the animals on the hot-plate. Control: Normal saline + Tween 80 (0.8%, v/v); Values are the mean ± SEM for 7 mice.

**Effects of thymoquinone on motor coordination**

 In rotarod test, 30 minutes after the administration of thymoquinone (40 mg/kg), the remaining time of animals on the rotating rod (time-on-rod) decreased significantly (p<0.05). Thymoquinone at the doses of less than 40 mg/kg (10 and 20 mg/kg) did not have any effects on motor coordination of mice in rotarod test (Table 6). Moreover, diazepam (4 mg/kg) as the positive control, significantly decreased the remaining time of mice on the rotating rod (p<0.001).

**Table 6.** Effect of thymoquinone on motor coordination of mice in rotarod test

**Drug (dose)**	**Time-on-rod (s)**	
	**30 min after the injection of thymoquinone**	**60 min after the injection of thymoquinone**
**Control**	300 ± 0.0	300 ± 0.0
**Diazepam** ** (4 mg/kg)**	3.7 ± 0.4 ***	5.2 ± 0.8 ***
**Thymoquinone** **(10 mg/kg)**	295.3 ± 2.3	294.9 ± 2.4
**Thymoquinone** ** (20 mg/kg)**	282.9 ± 5.1	283.3 ± 4.6
**Thymoquinone ** **(40 mg/kg)**	264.0 ± 11.7 *	272.9 ± 12.9

Thymoquinone and control were administered 60 and 30 min prior to the test. Values are the length of time each animal remained on the rotating rod (time-on-rod). Control: Normal saline + Tween 80 (0.8%, v/v); Values are the mean ± SEM for 7 mice;

* p<0.05,

*** p<0.001, as compared to control.

## Discussion

The results of the present study showed that thymoquinone attenuated the development of tolerance to analgesic effects of morphine. Thymoquinone also prevented the induction of physical dependence to morphine and attenuated the severity of the signs of withdrawal syndrome in morphine dependent mice. 

It has recently been reported that *N. sativa* oil prevented tolerance and dependence induced by morphine and tramadol in mice (Abdel-Zaher et al., 2010 ▶, 2011). Since thymoquinone is the major constituent of *N. sativa* oil, it seems that the inhibitory effects of *N. sativa* on opioid-induced tolerance and dependence arise from the beneficial effects of thymoquinone. 

In the present study, repeated administration of morphine over the course of 3 days, induced physical dependence in mice so that the injection of naloxone elicited the signs of withdrawal syndrome (Table 1). This protocol has been implemented in several previous studies to induce morphine tolerance and dependence in mice (Zarrindast and Torkaman-Boutorabi, 2003 ▶; Imenshahidi et al., 2007 ▶; Abdel-Zaher et al., 2010 ▶, 2011). 

The results of the present work showed that the pretreatment of animals with thymoquinone during the 3-day administration of morphine, attenuated the signs of withdrawal syndrome precipitated by naloxone on the fourth day (Table 1). Moreover, the administration of single doses of thymoquinone before the injection of naloxone on the fourth day, resulted in efficient reduction of the signs of withdrawal syndrome. The effect of both single and repeated administration of thymoquinone on physical dependence to morphine was dose-dependent so that the attenuation of the signs of withdrawal syndrome by thymoquinone at the dose of 40 mg/kg was significantly greater than that of 20 mg/kg (Tables 1, 2). The beneficial effects of thymoquinone on morphine dependence were comparable to that of diazepam (Tables 1, 2).

The results of this study showed that thymoquinone could also be efficacious against tolerance to analgesic effect of morphine. Following repeated administration of morphine over the course of 3 days, the pain threshold in animals decreased in hot-plate test. As it is shown in Table 3, the latency of nociceptive response in mice significantly decreased on the fourth day reflecting the diminution of morphine analgesia. However, pretreatment of animals with thymoquinone during the 3-day administration of morphine, dose-dependently prolonged the latency of nociceptive response in mice indicating the attenuation of tolerance to analgesic effect of morphine. Furthermore, the administration of single dose of thymoquinone (40 mg/kg) before the injection of the last dose of morphine on the fourth day, increased the pain threshold of animals in hot-plate test and preserved the analgesic effect of morphine (Table 4). The inhibitory effect of thymoquinone on morphine tolerance seems not to be related to its analgesic effects because none of the doses of thymoquinone produced any antinociceptive effect on their own (Table 5). 

Tolerance to opioid analgesics, defined as a loss of analgesic effect following repeated treatments such that a higher dose of the drug is required for equivalent effect. This may also lead to the opioid dependence and further complicate the process of pain treatment (Alldredge et al., 2013 ▶). μμμPasternak, 2001 ▶; Contet et al., 2004 ▶κμκμPark et al., 2006 ▶; Shippenberg et al., 2007 ▶). For example, it has been reported that TRK-820 and LPK-26 as κTsuji et al., 2000 ▶; Tao et al., 2008 ▶κκAbdel-Fattah et al., 2000 ▶). Accordingly, it is proposed that the agonistic effect of thymoquinone on κ

Various studies have also shown that some pharmacological effects of thymoquinone including antianxiety and anticonvulsant effects may be attributed to its stimulating effect on the central GABA_A_ receptors (Hosseinzadeh and Parvardeh, 2004 ▶; Hosseinzadeh et al., 2005 ▶; Gilhotra and Dhingra, 2011 ▶). It has been well documented that GABAergic system plays a crucial role in the development and expression of dependence and tolerance to opioid drugs. In support of this view, it has been reported that diazepam and midazolam as positive modulators on the benzodiazepine site in the GABA_A_ receptors and muscimol as a selective agonist of GABA_A_ receptors, prevent morphine dependence and suppress the signs of withdrawal syndrome in morphine-dependent mice (Tejwani et al., 1993 ▶; Suzuki et al., 1996 ▶; Tejwani et al., 1998 ▶; Zarrindast and Mousa-Ahmadi, 1999 ▶). Considering the effects of thymoquinone on GABAergic system, it seems that the inhibitory effects of thymoquinone on dependence and tolerance to morphine, may be related to its agonistic activity on GABA_A_ receptors. 

On the other hand, the role of calcium currents through voltage dependent calcium channels has been established in tolerance and dependence to opioids (Dogrul et al., 2002 ▶; Seth et al., 2011 ▶; Shimatani et al., 2014 ▶). For example, diltiazem and verapamil as L-type calcium channel blockers and mibefradil as T-type calcium channel blocker, prevented the development of tolerance to analgesic effects of morphine and suppressed the signs of morphine withdrawal syndrome (Dogrul et al., 2002 ▶; Seth et al., 2011 ▶; Shimatani et al., 2014 ▶). Some evidence indicated that thymoquinone reduces calcium influx through VDCCs in a variety of cells (Parvardeh and Fatehi, 2003 ▶, 2007). It is thus proposed that the beneficial effects of thymoquinone on dependence and tolerance to morphine seem to be caused, at least in part, by its inhibitory effects on VDCCs. 

Thymoquinone and its parent herbal medicine, *N. sativa*, are among the most well-studied antioxidant compounds. Many lines of studies have indicated that thymoquinone functions as a potent antioxidant and radical scavenger (Khan et al., 2012 ▶; Jrah-Harzallah et al., 2012 ▶, 2013). Several studies have suggested that oxidative stress and NO, might be involved in the development of dependence and tolerance to opioid analgesics (Doyle et al., 2009 ▶; Özek et al., 2003 ▶; Abdel-Zaher et al., 2013 ▶). In support of this view, various antioxidant agents and NOS inhibitors have been used to prevent dependence and tolerance to morphine and their beneficial effects has been reasonably well established. Recently, it has been reported that *N. sativa* oil attenuates tolerance and dependence to morphine and tramadol through inhibiting the activity of inducible NOS enzyme (Abdel-Zaher et al., 2010 ▶, 2011). These results are consistent with the findings of the previous studies indicating the inhibitory effects of thymoquinone on NOS enzyme (Gilhotra and Dhingra, 2011 ▶; Sayed, 2012 ▶). It is thus suggested that the antioxidant activity of thymoquinone together with its NOS inhibitory effect, may play an important role in the attenuation of dependence and tolerance to morphine.

As it is shown in Table 6, thymoquinone at the dose of 40 mg/kg, decreased the remaining time of animals on the rotating rod. Thus, it is possible that the impairment of motor coordination by the highest dose of thymoquinone (40 mg/kg) may have had some influence on behavioral responses of animals in both jumping and hot-plate tests. However, thymoquinone at the lower doses (10 and 20 mg/kg) did not have any effects on motor coordination of mice in rotarod test (Table 6). Thus, the effect of thymoquinone at the dose of 20 mg/kg on morphine dependence and tolerance, is not attributed to the impairment of motor coordination of animals.

Based on the results of this study, it can be concluded that thymoquinone attenuates dependence and tolerance to morphine in mice. The current study provides evidence for the effectiveness of applying thymoquinone to prevent tolerance to analgesic effects of morphine and to attenuate the acute signs of morphine withdrawal syndrome. 
